# A dual mobile eye tracking study on natural eye contact during live interactions

**DOI:** 10.1038/s41598-023-38346-9

**Published:** 2023-07-14

**Authors:** Florence Mayrand, Francesca Capozzi, Jelena Ristic

**Affiliations:** 1 Department of Psychology, McGill University, 1205 Dr Penfield Avenue, Montreal, QC H3A 1B1 Canada; 2 Department of Psychology , Université du Québec à Montréal (UQAM), Montreal, Canada

**Keywords:** Human behaviour, Psychology

## Abstract

Human eyes convey a wealth of social information, with mutual looks representing one of the hallmark gaze communication behaviors. However, it remains relatively unknown if such reciprocal communication requires eye-to-eye contact or if general face-to-face looking is sufficient. To address this question, while recording looking behavior in live interacting dyads using dual mobile eye trackers, we analyzed how often participants engaged in mutual looks as a function of looking towards the top (i.e., the Eye region) and bottom half of the face (i.e., the Mouth region). We further examined how these different types of mutual looks during an interaction connected with later gaze-following behavior elicited in an individual experimental task. The results indicated that dyads engaged in mutual looks in various looking combinations (Eye-to-eye, Eye-to-mouth, and Mouth-to-Mouth) but proportionately spent little time in direct eye-to-eye gaze contact. However, the time spent in eye-to-eye contact significantly predicted the magnitude of later gaze following response elicited by the partner’s gaze direction. Thus, humans engage in looking patterns toward different face parts during interactions, with direct eye-to-eye looks occurring relatively infrequently; however, social messages relayed during eye-to-eye contact appear to carry key information that propagates to affect subsequent individual social behavior.

## Introduction

Much of human social communication occurs nonverbally, with visual signaling from the eyes constituting a vital component of this communicative dynamic. Social visual signaling is fundamental for human social function^1^. In natural interactions, we encode information conveyed by the eyes and faces to determine focus of attention, mental states, behavioral intentions, and psychological states^2,3^. In addition, we simultaneously signal this information back to others through our own gaze^4,5^ and facial expressions^6,7^. Thus, in social communication, we both perceive social cues from others and signal social information back to them with our eyes^4^. This dual function of human eye gaze is critical for facilitating interactive behaviors such as turn-taking^8–10^, social perception such as leadership or social status^5,11–13^, as well as joint attention and cooperation^14,15^. The present study examined the characteristics of mutual looks during natural interactions in live two-person dyads and assessed how different types of looking behaviors experienced at a group level linked with later gaze following in individual group members.

Humans preferentially attend to and look at social stimuli such as others’ eyes and faces^16,17^. During real-life interactions, social cues from the eyes are not only passively perceived but also reciprocated via mutual looks. In natural interactions, mutual looks are thought to signal social interest and serve as a foundation for shared attention^18^ and prosocial behavior^18–20^. As a salient example of mutual looking behavior, eye-to-eye contact is preferred from early on in development^21^, associated with detecting communicative intent^22,23^, and eliciting positive emotions in observers^24^. Relatedly, gaze following, or the ability to orient attention in the direction of another person’s gaze, is also thought to depend on the social information conveyed by the eyes. Indeed, eyes are theorized to carry both mentalistic and directional information^25,26^.

While mutual looks are considered to represent one of the fundamental behaviors for relaying social messages nonverbally, there is surprisingly little knowledge about the prevalence and characteristics of this behavior. One reason for this knowledge gap is past technical limitations in measuring eye movements during real-life interactions due to the lack of high-speed mobile eye-tracking solutions. For example, Capozzi and Ristic^25^ used the patterns of looking hand-coded from concealed eyeglasses cameras which participants wore during live interactions to define and extract gaze dynamics in live interacting three-member groups (see also^11^). Using this method, the authors found that mutual looking (which they operationalized as reciprocal face-to-face looking between two group members and labelled as mutual gaze) occurred about 30% of the interaction time, with the higher amounts of mutual looks during the interaction relating to higher perceptions of leadership within the group^25^. Using similar equipment, Hayward et al.^27^ estimated that dyads engaged in mutual looks about 80% of the time during live interactions, while others found that when individuals are interacting with others in natural settings, they rarely engaged in mutual looks, only about 10% of time^28–30^. Finally, using a stationary setup with dual head-mounted eye trackers, in which the two participants sat in physically different locations and interacted with each other live via a video feed, Haensel, Smith, and Senju^31^ examined cultural influences on the prevalence of direct eye contact in East Asian (EA) and Western Caucasian (WC) dyads, and found higher proportions of eye-to-eye looks in EA relative to WC groups. However, since in this examination the participants were not free to behave naturally and the fixations were not examined as a function of face parts, it remains unknown whether these reciprocal mutual looks during social interactions reflect direct eye-to-eye contact, or also occur when we reciprocally look at other parts of the face or the face as a whole. Similar concerns apply to Capozzi and Ristic and Hayward et al. studies in which the authors could not extract fine-grained temporal or spatial information about looking behaviors.

There are also relatively few studies which have examined the links between eye contact experienced during live interactions and later gaze following behavior. Early studies had suggested that engaging in direct gaze was a precursor for later gaze following^23^ in that gaze following only occurred when an averted gaze shift was preceded by direct eye contact indicating communicative intent. More recently, interactive studies have investigated how different types of mutual looks experienced during real-life interactions connected with later gaze-following as assessed using standard gaze-cuing procedures^32^. For example, Hayward et al.^27^ tested whether the amount of direct gaze contact experienced during a dyadic interaction predicted individual participants’ magnitude of gaze following elicited by schematic face gaze cues in a subsequent gaze following task. While both direct eye contact and gaze following occurred reliably, no significant relationship emerged between them. Similarly, relating the amount of mutual looks experienced during a three-person interaction with the magnitude of gaze following elicited in the later individual gaze following, Capozzi and Ristic^25^ found no relationship between the amount of mutual looking during a group interaction and the magnitude of gaze following elicited by the partner’s image in a later gaze cuing task. However, and as discussed above, the measure of mutual looking was not based on a precise region of interest analysis of eye data, and thus was not specific to direct eye contact.

In the present study, we used dual mobile TOBII eye tracking eyeglasses to measure and characterize mutual looks during natural dyadic interactions. This technology allowed us to measure looking behavior while participants remained unconstrained, physically present within the same space, and freely interacting. It also allowed us to perform a more nuanced and synchronized spatial and temporal analysis of looking to assess the prevalence of looking directed to the top half of the face (i.e., the Eyes) and those directed to the bottom half of the face (i.e., the Mouth) in both interactive partners. To understand if direct eye-to-eye contact between partners was needed for reliable gaze following to occur in later assessments, we also examined how looking behaviors during the interaction related to individual magnitudes of gaze following elicited by gaze cues of interactive partners^25^.

Based on past work [e.g.,^27^] we expected to find direct eye-to-eye looking patterns to be prevalent during the interactions, with other face-directed combinations of mutual looking behaviors (i.e., Eyes-Mouth, Mouth-Mouth) as well as non-mutual looking behaviors (i.e., unidirectional looking not directed to the partner’s face) occurring less frequently. Furthermore, if gaze following reflected the reading of social information from the eyes, we expected to find a positive correlation between the amount of eye-to-eye mutual looks experienced during the interaction and the magnitude of the gaze following measured in the later experimental task.

## Methods

### Participants

A total of 30 participants forming 15 mixed-gender dyads were recruited for the study (25 females, 5 males, mean age = 20.3, age range = 18–24; N = 11 female-female dyads; N = 5 male–female dyads). Data from five participants were lost due to low eye tracking data quality (three dyads), as any participant (and consequently the dyad) from whom the TOBII gaze sampling data quality was below 70% was excluded. Two dyads were excluded due to missing gaze cuing task data and GLI questionnaire data. Hence, data from 14 participants forming 7 mixed-gender dyads were analyzed (12 females, 2 males, 2 mixed-gender dyads, mean age = 20.21, SD = 1.85). This final sample approximates typical sample sizes in this type of design^25^, which range from 13 groups in Capozzi and Ristic^25^ and 14 groups in Capozzi et al.^11^ to 20 dyads in Haensel et al.^31^. Sensitivity analysis of the current data indicated that the power between 0.80 and 0.90 for the Alpha level of 0.05 would reflect the effect size ranging from *f* = 0.54 to 0.62, which agrees with effect sizes reported by Haensel et al.^31,33^. Participants were recruited from the McGill Psychology Human Participant Pool and received course credits. No participants knew one another, and all provided informed consent. All experimental protocols were approved by the McGill University’s Research Ethics Board, and all methods were performed in accordance with the relevant guidelines and regulations. Informed consent for publication of identifying information/images in an online open-access publication was obtained for all individuals illustrated in this manuscript. No information or images identifying study participants are used.

### Apparatus, stimuli, and design

**Figure 1:**
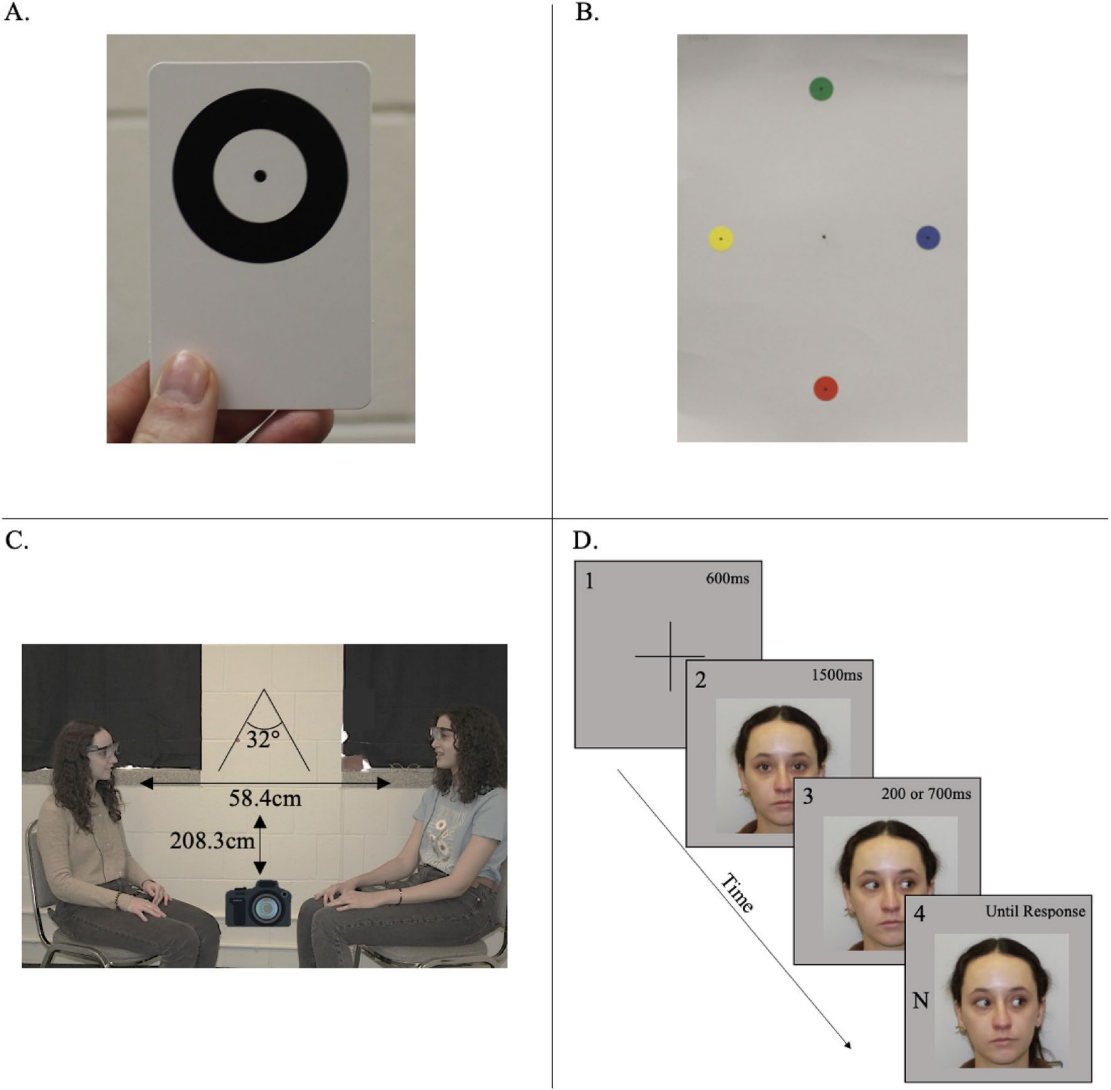
(**A**) Tobii Calibration Card. (**B**) Second calibration display. (**C**) Illustration of the interaction set up. Participants sat on chairs placed 58 cm apart and positioned at an outwards angle of 32 degrees to face a wide screen camera which was mounted on a tripod that was 208.3 cm away from the chairs at the height of 116.8 cm. (**D**) Illustration of an individual Gaze Cuing Task. After the 600 ms presentation of a fixation cross (1), the face of the fellow participant appeared looking straight ahead for 1500 ms (2). The face then shifted their gaze to the left or right (3). After 200 or 700 ms, the response target appeared in the gazed-at (valid, like in this example) or not gazed-at (invalid) direction (4). Intertrial interval was 600 ms. Please note that the images used here are for illustrative purposes only and do not depict the participants/images used in the study.

Participants completed a natural interaction in pairs and a computerized gaze cuing task individually. Figure 1C illustrates the interaction room setup. Participants sat on chairs placed 58 cm apart and positioned at an outwards angle of 32 degrees to face a wide screen Cannon T2i/EOS550D SLR camera. The camera was mounted on a tripod positioned 208.3 cm away from the chairs’ center at the height of 116.8 cm. The camera filmed the entire interaction at the pixel resolution of 1920 × 720 and 30 frames per second (fps).

During the interaction, both participants wore Tobii Pro ETG 2 remote eye-tracking eyeglasses. The eye-tracking glasses have a front-facing camera that captures the participant’s visual field at the pixel resolution of 1920 × 1080 and 25fps and records their gaze with a sampling resolution between 50 and 100 Hz, depending on conditions such as angles. To increase gaze sampling, participants were advised not to lean forward during the interaction. Four inward-facing eye-tracking sensors, two for each eye, record the participant’s eye movements and orientation. The eye-tracking glasses rest on the bridge of the participant’s nose. An adjustable strap ties around the back of the participant’s head to secure the glasses in place and prevent slipping.

In the individual gaze cuing task, illustrated in Fig. 1D, color photographs of the dyad partner’s face showing center, left, and right gaze served as stimuli. Photographs of participants looking straight ahead were taken at the start of the session (see "Procedure") and were modified using Adobe Photoshop to display averted left and right gaze direction. Each photograph measured 10.5 cm (10 degrees of visual angle) by 11.4 cm (10.85 degrees of visual angle).

Response targets were capital letters “H” or “N” measuring 2° × 3° of visual angle. The task was controlled by a Mini Mac computer connected to a 16-inch CRT color monitor. Participants viewed the stimuli from an approximate distance of 60 cm. The experimental sequence was controlled by Experiment Builder (SR Research).

### Design

The study was a repeated measures design with participants first completing the interaction part and then individual gaze cuing task in that order. To ensure equality in terms of group social composition, participants also completed an adapted General Leadership Impressions scale (GLI^34,35^ which included an additional question about perceived interactive partner’s similarity (i.e., “How similar to yourself do you perceive this individual?”). Participants rated each item using a Likert scale (1/Lowest to 5/Highest). The questionnaire was filled out after the interaction and before the individual task. All dyads reported equivalent GLI (M = 3.78, SD = 0.65) and similarity scores (M = 3.36, SD = 0.86).

### Dyadic Interaction

The interaction was stimulated using a survival task^36^. Here, participants are presented with one of two imaginary survival scenarios (“Winter” or “Desert”) and are asked to arrive at a ranking of a list of items (e.g., compass, hand axe, chocolate bar) in the order of their usefulness for the group survival. The experimenter repeated the 12-item list twice before leaving the room. The interaction ended either when *(i)* the dyad completed the task and went to retrieve the experimenter who was waiting outside the room or *(ii)* after 20 min had elapsed, whichever came first. At the end, the dyad reported their ranking of items to the experimenter, who recorded their answers.

### Individual Gaze Cuing Task

The individual task was a standard gaze cuing task^32,37^, with the example stimuli and trial sequence illustrated in Fig. 1D. Here, Response Times (RTs) are measured for targets that appear at gazed-at relative to not gazed-at locations. Critically, and adapting the standard procedure, in the present study, the images of the gazing faces were the photographs of the interactive partners from the dyadic interaction^25^. This ensured a linking of the group interaction with individual gaze following behavior.

The gaze-cuing task manipulated three within-subject factors. Gaze Direction (Left, Right) varied the gaze direction between left and right. Target Location (Left, Right) varied the location of the target between the left or right of the face. Congruent gaze direction and target locations (e.g., left gaze followed by left target) made up valid or cued trials. Incongruent gaze direction and target locations (e.g., left gaze followed by right target) made up invalid or uncued trials. Finally, the time between the presentation of the face and the presentation of the target varied between 200 and 700 ms. This Stimulus Onset Asynchrony (SOA) interval is commonly manipulated to capture the time course of gaze following behavior^38,39^. Gaze direction, target location, and SOA varied equally and equiprobably, such that the target was equally likely to appear on either left or right side of fixation regardless of gaze position and following the short or the long-time delay.

## Procedure

Upon arrival, and after completing the informed consent, participants were instructed about the nature of the tasks and eye movement recordings. Then, their pictures were taken, and the dyad was brought into the room and fitted with the Tobii Pro Glasses.

Two calibration procedures were performed. First, to confirm tracking, participants held Tobii’s calibration card showing a black dot at the center at arm’s length and were asked to look at the center dot (Fig. 1A). This allowed the experimental software to confirm that the eye-tracking glasses accurately measured the participant’s eye movements. Second, to understand the spatial precision of eye position tracking, we additionally presented participants with a different calibration card which showed 5 separate circles (Fig. 1B). In this calibration procedure, participants were asked to stand approximately 100 cm away from this card and to fixate on each circle for 3 s, by moving their eyes from center dot, to top, then to right, bottom, left, and back to center dot. Then, dyads were seated on chairs and given instructions for the survival task.

Following the interaction, participants were given a 5-min break and were then escorted into a different space to complete the computerized gaze-following task individually. Trials began with a 600 ms presentation of a central fixation cross. Then, the face looking straight ahead was shown for 1500 ms, after which the face image looking either left or right was shown. The response target appeared 200 or 700 ms following the onset of the averted gaze cue. Participants were instructed to identify the target letter (H or N) quickly and accurately by pressing either the ‘b’ or ‘v’ buttons on the keyboard, which were marked by blue and yellow stickers. Response target-key color pairings were counterbalanced across participants. Both the face cue and the target remained on the screen until a response was made or until 2500 ms had elapsed, whichever came first. If participants did not respond or responded incorrectly, a feedback tone was played. Inter-trial interval was 600 ms.

Participants were informed that gaze direction did not predict the target's location and instructed to maintain central fixation. The task consisted of 288 experimental trials divided over 4 blocks. Sixteen practice trials were run at the start. The entire study took about 120 min to complete.

## Results

### Data and Measures

Eye tracking data were analyzed using Tobii Pro Lab Analyzer software, version 1.142. For each dyad, we identified an approximately five-minute long Time of Interest (TOI) window (M = 274.8 s, Range: 180–300 s) during the middle and second part of the interaction (starting anywhere between minutes 5 and 16), which allowed for the interaction to develop naturally, in which TOBII eye gaze sampling accuracy continuously exceeded 70% for each participant (M = 78.37%, Range: 56.76–91.02%). These TOI windows were the first instances of continuous high gaze sampling after the 5-min mark for each recording. This temporal selection was done to ensure that the data were of high enough quality as well as to streamline the eye movement analyses, which depended on hand-coding dynamic Regions of Interest (ROI) for each participant and video frame. This coding approach is on par with past studies which utilized dynamic ROI computation^25,27^.

### Spatial accuracy

**Figure 2:**
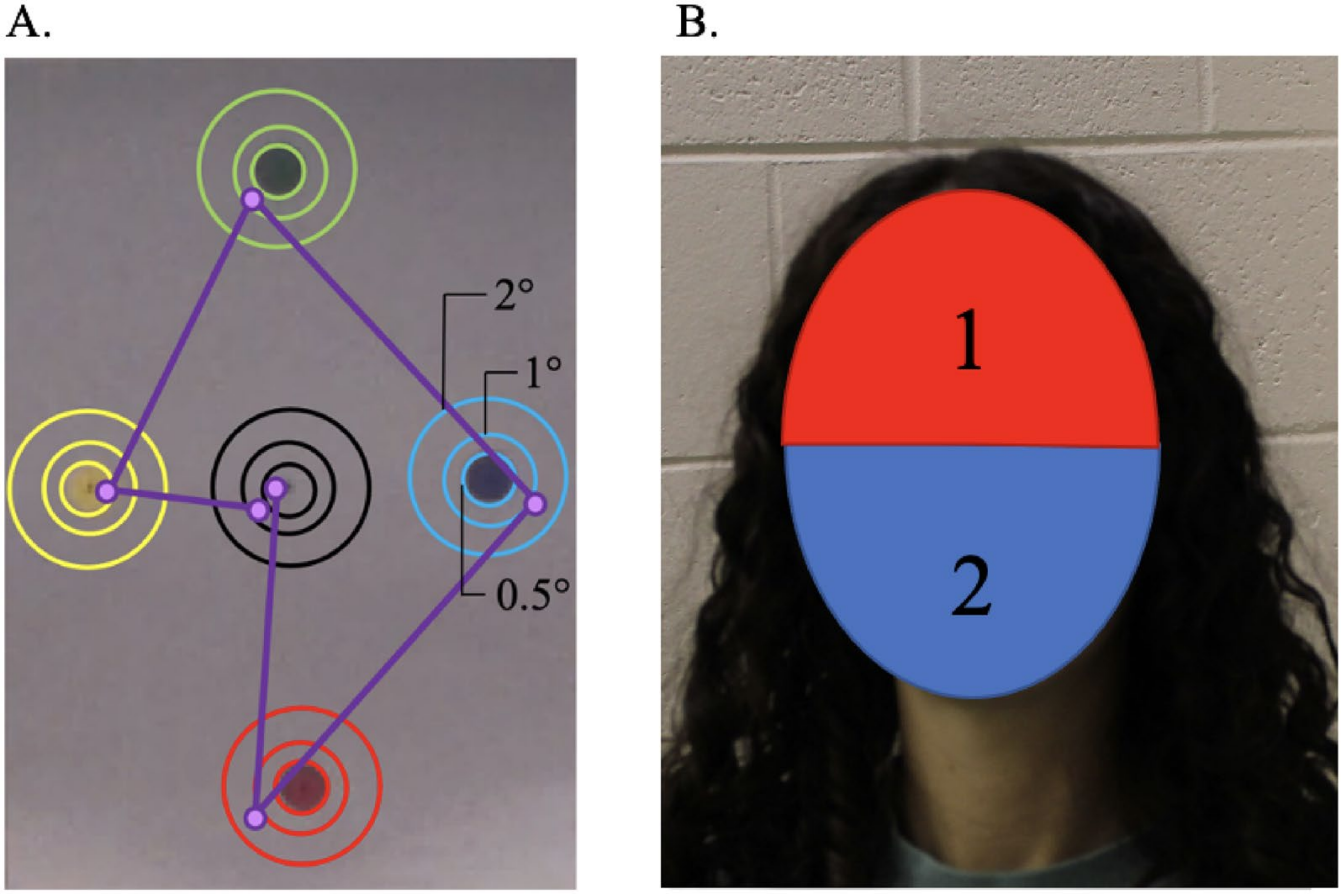
(**A**) Calibration Sheet with ROIs. The ROIs measured (1) 0.5 degrees of visual angle, (2) 1 degree of visual angle, and (3) 2 degrees of visual angle with trajectory of fixations. (**B**) Illustration of the dynamic face ROIs: (1) Eye Region; (2) Mouth Region. Note that images are not drawn to scale.

To examine the spatial accuracy of eye movements, and to ensure resolution size of the ROIs, we also examined fixations from the second calibration procedure by measuring the proportion of fixations falling on each calibration dot center (spanning 0.5°) and the surrounding 1° and 2° areas around the dot’s center. Figure 2A shows the calibration areas of interest and an example fixation path from one participant. We tabulated fixations by coding fixations as a hit when they fell within the spatial bounds of one of three calibration areas. Then, all hits for each participant as a function of calibration area were summed. This indicated 85% spatial accuracy for areas of 2°; 55% spatial accuracy for areas of 1°; and 27% spatial accuracy for areas of 0.5°. Thus, we designed all ROIs to span more than 2° of visual angle.

### Regions of Interest (ROI)

To examine eye movements during the interaction, we defined two dynamic regions of interest (ROIs) for each participant: *(i) Top face* encapsulating the upper portion of each participant’s face from the nose bridge to the top of the forehead (i.e., Eye-region ROI), and *(ii) Bottom face* encapsulating the lower portion of each participant’s face from the nose bridge to the end of the chin (i.e., Mouth-region ROI). Each ROI was manually added to the recording and then manually adjusted from frame to frame to include the same spatial area. ROIs varied in sizes since they captured each participant’s individual face and the remaining image area. On average, ROIs measured 3.82° by 3.16° of visual angle.

### Looking Behaviors

Two types of looking behaviors were defined—Mutual and Non-mutual looks. Mutual looks were defined as behaviors in which each partner was looking at one of the predefined face ROIs, thus the partners looked at each other’s faces. Non-mutual looks were defined as gaze behaviors in which one or both partners did not look at one of the predefined face ROIs, thus one or both partners looked away from the other partner’s face.

### Mutual looks

**Figure 3:**
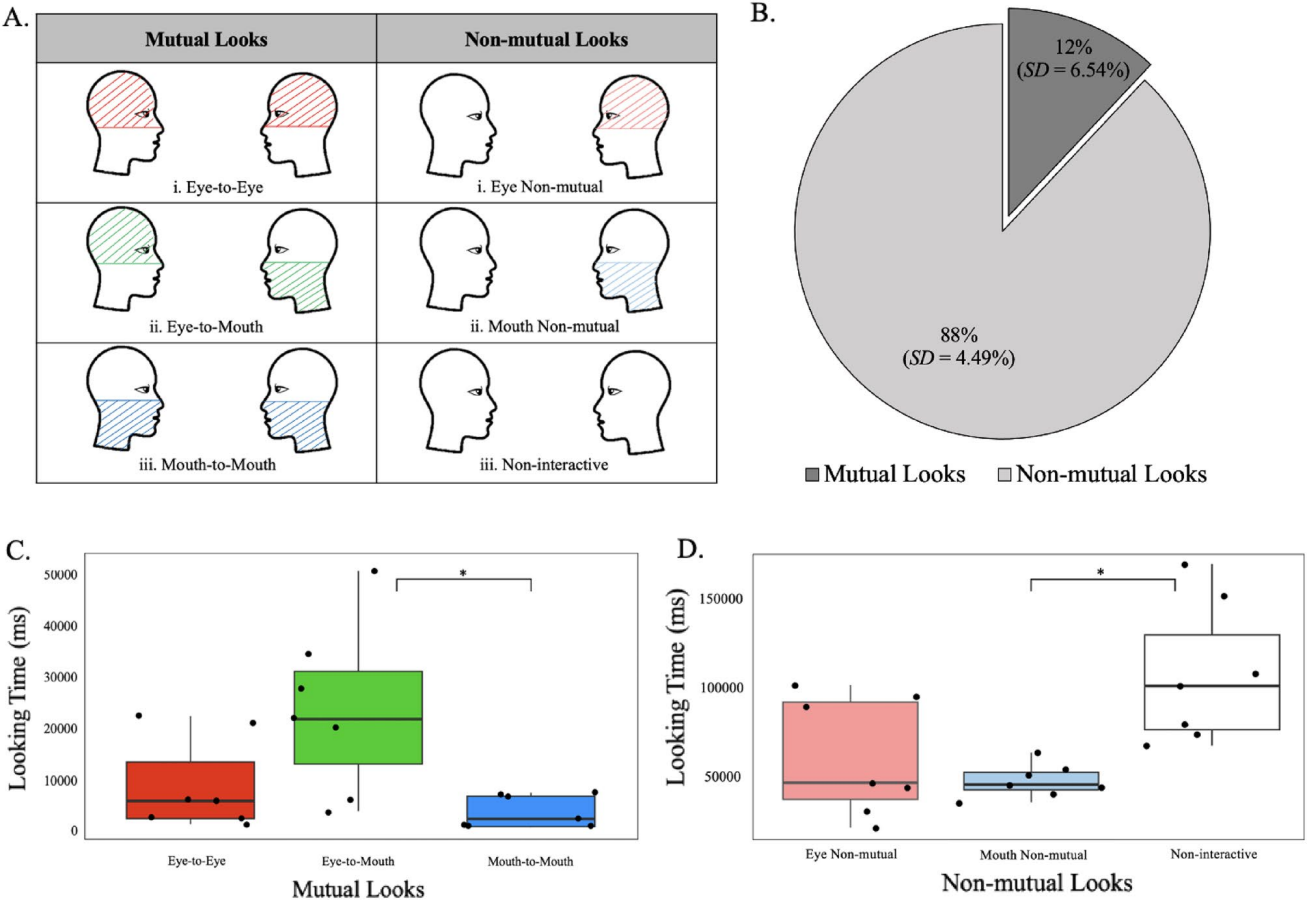
(**A**) Types of Mutual (Eye-to-Eye, Eye-to-Mouth, and Mouth-to-Mouth) and Non-mutual (Eye, Mouth, Non-interactive) looking behaviors. Colored areas depict ROIs where gaze is directed. (**B**) Proportion of interaction time spent in Mutual and Non-mutual looks. (**C**) Average and individual participant looking times for Mutual looks as a function ROI combination. (**D**) Average and individual participant looking times for Non-mutual looks as a function ROI combination. Error bars represent 95% Confidence Intervals (CI); *p < 0.05.

Mutual looks reflected instances in which both partners simultaneously looked at one of the face ROIs at their partners face, i.e., they engaged in mutual gaze. Figure 3A shows three possible combinations of mutual looks: *(i) Eye-to-Eye* in which participant A looked at the top half of participant B’s face, while at the same time participant B looked at the top half of participant A’s face; *(ii) Eye-to-Mouth* in which participant A looked at the bottom half of their partner’s face, and their partner looked at the top half of their face (and vice versa—conditions in which participant B looked at the bottom half of their partner’s face, and partner A looked at the top half of their face); and *(iii) Mouth-to-Mouth* in which participant A looked at the bottom half of participant B’s face, and participant B looked at the bottom half of participant A’s face.

### Non-mutual looks

Non-mutual looks were defined as any other gaze behavior in which one or both partners did not look at one of the predefined face ROIs. Figure 3A shows three possible types of non-mutual looks *(i) Eye Non-mutual* looks reflected conditions in which one participant looked at the top ROI of their partner’s face while the other participant looked at no face ROI; *(ii) Mouth Non-mutual* looks reflected conditions in which one participant looked at the mouth ROI of their partner’s while the other partner looked elsewhere, and *(iii) Non-interactive* looks in which neither partner looked at any of the predefined face ROIs, reflecting periods of time when both participants simultaneously looked away from the face of their partner.

## Analyses

We analyzed data on both dyad and individual levels. To understand the amount of interaction time spent in different types of mutual looks on a dyad level, we first tabulated the time spent in mutual and non-mutual looking behaviors for each dyad. This was accomplished by measuring and aggregating the dwell time of fixations that fell within each of the predefined ROIs for each participant and by synchronizing the temporal recording of the data within each dyad. We then separated these measures by the time spent in different ROI combinations for Mutual and Non-mutual looks. Finally, we divided the time spent in these combinations by the total amount of TOI (i.e., interaction) time for each dyad. These analyses addressed the hypotheses about the prevalence of mutual looks, and specifically eye-to-eye direct contact in natural interactions.

On an individual level, we analyzed the data from the individual gaze cuing task, and then, using regression analyses, related the prevalence of mutual and non-mutual looks during the interactions as a function of ROI combination to individual participants’ magnitude of gaze following. This analysis addressed the hypothesis about relating mutual looks at a group level with gaze following at the individual level.

### The prevalence of mutual and non-mutual looks

First, we examined the interaction time dyads spent in mutual and non-mutual looking. Figure 3B shows that dyads spent the most time, 88% (*SD* = 4.49%), engaging in non-mutual looks, and only 12% (*SD* = 6.54%) of interaction time engaging in mutual looks. This is a statistically significant difference (*t*(13) = 24.353, *p* < 0.001, two tailed, paired).

### Mutual looks Regions of Interest (ROI) Analyses

Next, we examined whether the time spent in mutual and non-mutual looking behaviors varied as a function of ROI looking combinations. This analysis was performed at dyad level, since the data from individual participants were not independent. Two one-way repeated measures ANOVAs were run, one for mutual and one for non-mutual looking behaviors. It is important to note that dyads were not constrained in the patterns of looking and thus spending more time in one type of ROI combination did not necessitate spending less time in a different ROI combination. Dyads were also unconstrained in the amounts of time spent in overall mutual and non-mutual looking behaviors. Hence, the different gaze behaviors were independent from one another.

The first ANOVA examined the time dyads spent engaging in three possible looking combinations (i.e., Eye-to-Eye, Eye-to-Mouth, and Mouth-to-Mouth; Fig. 3A). The data, illustrated in Fig. 3C, showed that dyads spent significantly more time engaging in Eye-to-Mouth looking relative to the other two ROI combinations, *F*(2, 12) = 7.215, *p* = 0.009, *MS* = 771,273,176, *η*_*p*_^*2*^ = 0.546. Follow-up paired t-tests showed that dyads spent the most time engaging in Eye-to-Mouth looking (*M* = 24,135.71, *SD* = 16,444.23) relative to Mouth-to-Mouth (*M* = 4005.71, *SD* = 3209.54), *t*(6) = 3.166, *p* = 0.019, but not to Eye-to-Eye (*M* = 8910.00, *SD* = 9123.11), *t*(6) = − 2.389 *p* = 0.054, looking combinations. Time spent in Mouth-to-Mouth and Eye-to-Eye looking behaviors did not differ, *t*(6) = 1.506, *p* = 0.183.

The second ANOVA examined the time dyads spent engaging in non-mutual looks as a function of ROI (Eye Non-mutual, Mouth Non-mutual, and Non-interactive). The data, plotted in Fig. 3D showed that participants spent the most time in the Non-interactive looking pattern, in which both participants did not look at their partner’s face ROIs, *F*(2, 12) = 6.340, *p* = 0.013, *MS* = 6,890,000,000, *η*_*p*_^*2*^ = 0.514. Follow up paired t-tests indicated that dyads spent significantly more time in the Non-interactive looking pattern (*M* = 110,357.14, *SD* = 39,666.15) relative to Mouth Non-mutual (*M* = 50,722.86, *SD* = 9250.73), *t*(6) = 4.106, *p* = 0.006), but not to Eye Non-mutual (*M* = 63,638.57, *SD* = 33,328.56), *t*(6) = − 2.055, *p* = 0.086), which did not differ, *t*(6) = 0.904, *p* = 0.401.

Overall, the examination of the different looking patterns during the interactions indicated that dyads spent the majority time (i.e., 88% of time) engaging in non-mutual looks, in which one or both partners did not look at the face of the other partner. During the remaining 12% of time, dyads engaged in mutual looks, with Eye-to-Mouth looking patterns the most prevalent relative to Eye-to-Eye and Mouth-to-Mouth looking patterns. Thus, mutual looks towards the face appear to occur infrequently, with Eye-to-Eye contact occurring in only about 3.5% of the interaction time.

We are mindful of the possible limitations that our sample size exerts on the interpretation of data. To potentially mitigate those limitations, we have performed an additional analysis, which included two additional dyads who achieved proper eye tracking data accuracy but were excluded due to missing individual components—cuing task and GLI questionnaire. The analysis of the eye tracking data with 18 participants (i.e., 9 dyads) confirmed the main results. As before, dyads spent the most time in Eye-to-Mouth ROI combination relative to the other two ROI combinations, F(2, 16) = 7.430, p = 0.005, MS = 732,759,478, ηp2 = 0.482, with more time in Eye-to-Mouth looking exchanges (M = 21,390.00, SD = 15,332.00) relative to Mouth-to-Mouth (M = 3504.44, SD = 2970.00), t(8) = 3.510, p = 0.008, but not to Eye-to-Eye (M = 10,365.56, SD = 8422.70), t(8) = − 1.953, *p* = 0.087, looking exchanges, which significantly differed, t(8) = 2.438, p = 0.041. The ANOVA on Non-mutual looking once again found that participants spent the most time in the Non-interactive looking pattern, F(2, 16) = 11.563, p = 0.001, MS = 9,972,000,000, ηp2 = 0.591, with dyads engaging significantly more in Non-interactive looking (M = 114,976.67, SD = 35,707.87) both relative to Eye Non-mutual (M = 69,015.56, SD = 30,856.48), t(8) = 2.631, p = 0.03, and Mouth Non-mutual conditions (M = 50,287.78, SD = 8058.00), t(8) = 5.558, p = 0.001, which did not significantly differ, t(8) = 1.616, p = 0.145. Thus, the main result held when additional participants were included in the analyses.

### Individual Gaze Cuing task

Mean correct interparticipant RTs were analyzed using a two-way repeated measures ANOVA with Cue-target congruency (Congruent; Incongruent) and SOA (200, 700 ms) included as variables. As expected and replicating a large volume of past work (e.g., Friesen & Kingstone, 1998), gazed-at, congruent targets were identified faster than non-gazed-at, incongruent targets, *F*(1,13) = 11.94, *p* = 0.004, *MS* = 1449.936, *η*_*p*_^*2*^ = 0.479. There was a main effect of SOA, *F*(1,13) = 9.401, *p* = 0.009, *MS* = 2856.857, *η*_*p*_^*2*^ = 0.420, and the interaction between SOA and Cue-target congruency was non-significant, *F*(1,13) = 1.23, *p* = 0. 287, *MS* = 263.96, *η*_*p*_^*2*^ = 0.087. Analyses of accuracy indicated that overall participants were more accurate at identifying gazed-at targets than non-gazed-at targets, *F*(1,13) = 9.682, *p* = 0.008, *MS* = 0.008, *η*_*p*_^*2*^ = 0.42. A main effect of SOA and the interaction between SOA and Cue-target congruency were not significant, F(1,13) = 0.039, *p* = 0. 846, *MS* = 0.00004, *η*_*p*_^*2*^ = 0.003; F(1,13) = 1.736, *p* = 0. 210, *MS* = 0.001, *η*_*p*_^*2*^ = 0.118.

### Regression analyses

Two separate multiple regression analyses were conducted to examine whether the prevalence of mutual and non-mutual looks during the dyadic interaction related to later gaze following at the individual level. The models examined whether the variability in looking time to different ROI patterns found during mutual and non-mutual looking behaviors at the dyad level predicted the magnitude of gaze cuing at the individual level. The gaze cuing magnitude was calculated by subtracting congruent from incongruent RT for each participant.

The first model used the individual time spent in mutual looks ROI combinations of Eye-to-Eye, Eye-to-Mouth, and Mouth-to-Mouth as predictors of the magnitude of the individual participants’ magnitude of the gaze cuing. This model overall was significant, *R*^*2*^ = 0.582, *F*(3,10) = 4.637, *p* = 0.028, indicating that more time spent in mutual looks during the interaction was a reliable predictor of the higher magnitude of individual participants’ gaze following later.

**Figure 4:**
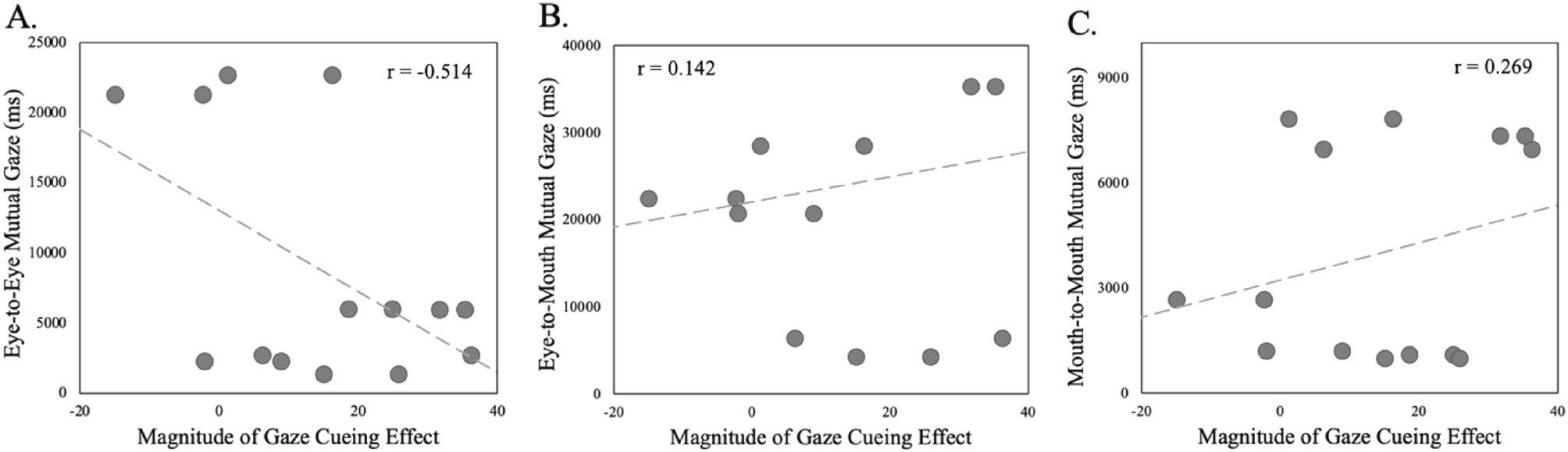
Scatterplots depicting the relationship between the time spent in (**A**) Eye-to-Eye, (**B**) Eye-to-Mouth, and (**C**) Mouth-to-Mouth interactive ROI combinations shown on the y-axis and individual participants’ magnitude of gaze following plotted on the x-axis.

Specifically, amount of time spent in Eye-to-Eye and Mouth-to-Mouth looking combinations were each a reliable predictor of the later gaze following magnitudes (Eye-to-Eye, *β* = − 0.764, *t*(13) = − 3.414, *p* = 0.007; Mouth-to-Mouth, *β* = 0.528, *t*(13) = 2.426*, p* = 0.036; Eye-to-Mouth *β* = 0.33, *t*(13) = 1.562, *p* = 0.149). Figures 4A-C show the scatterplots depicting the relationship between the time spent in each of the interactive ROI combinations (x-axis) and individual participants’ magnitude of gaze following (y-axis).

The second model examined the link between the time spent in non-mutual ROI combinations with individual time spent in Eye Non-mutual, Mouth Non-mutual, and Non-interactive looking behaviors entered as predictors of the individual magnitude of the gaze cuing. The overall model was reliable, *R*^*2*^ = 0.681, *F*(3,10) = 7.102*, p* = 0.008, with more time spent in Non-interactive looking, *β* = − 0.578, *t*(13) = − 3.001*, p* = 0.013, being a reliable predictor of a smaller gaze cuing effect at the individual level. More time spent in Mouth Non-mutual behavior was a reliable predictor of a larger gaze cuing effect, *β* = 0.611, *t*(13) = 3.142*, p* = 0.01, as we also found for the mutual looking combinations. Importantly, the time spent looking at the eyes in the non-mutual ROI arrangements was not reliably associated with individual gaze cuing magnitude, *β* = − 0.360, *t*(13) = − 1.788, *p* = 0.104.

An examination of the collinearity statistics indicated no significant relationships between predictor variables for the regression examining the relationship between gaze cuing magnitude and mutual looking behaviors (all *VIF*s < 1.1199) and marginal levels of collinearity for the regression examining the relationship between gaze cuing and non-mutual looking behaviors (all *VIFs* < 5.626). The latter overlap may not be surprising since participants spent the overwhelming amount of time engaging in non-mutual looking behaviors.

Thus, the results of the regression analyses indicated that while constituting the minority of the interaction time, the time spent in mutual direct eye-to-eye contact was positively related to the larger magnitude of gaze following elicited by interactive partner’s gaze cues. For non-interactive looking patterns, more time spent not looking at the partner was significantly related to the smaller gaze cuing effect. As such, it appears that mutual interactive exchanges are a critical factor linking social dynamics at the group and individual levels.

Curiously, our data from the mutual and non-mutual regressions both indicated that looking at the mouths of interactive partners, either in mutual or non-mutual arrangements, constituted a significant predictor of individual gaze following magnitudes, such that longer looking times at the mouth were associated with higher gaze cuing magnitudes. This may reflect looking patterns during speaking. That is, when someone is speaking, they might glance at, or check on, their interactive partner’s face in order to confirm understanding^8^. At the same time, the partner who is listening, might look to the speaker’s mouth in order to anticipate or follow what they are saying^40^. To probe into this result, Pearson correlations were used to examine how the ratio of speaking to listening time for each participant related to their ROI looking times. Speaking time was coded by naïve observers who listened to the recordings and marked each timeframe of the video TOI in which a participant was speaking. Then, the ratio of speaking to listening time was calculated by dividing the amount of time (in ms) each participant spoke by the amount of time (in ms) each participant spent listening. For mutual looking ROI conditions, correlation data revealed that participants looked at the mouths of their partners more when partners spoke relative to when they themselves spoke, *r*(12) = 0.584, *p* = 0.014. This suggests that looking at interactive partners while they speak may contribute to magnitudes of later gaze following. All other correlations between the ratio of speaking and listening and the remaining mutual and non-mutual looking behaviors (all *r*s < 0.337, *p*s > 0.239) were not reliable. These results dovetail with the finding indicating that dyads spent the most time spent in Eye-to-Mouth looks during interactions. Thus, the patterns of Eye-to-Mouth looking may allow dyads to simultaneously attend to the facial regions of one another to maximize available visual information^41^. Future higher powered studies in which looking-while-listening and looking-while-speaking are manipulated systematically are needed to understand the relationship between speaking time, mutual gaze, looking patterns, and later social function^42^.

## Discussion

Using dual mobile high-speed eye tracking, we examined the prevalence of mutual and non-mutual looking behaviors during natural dyadic interactions. Our data indicated that participants engaged in both mutual and non-mutual looks, with significantly more time spent in non-mutual (i.e., looking away from the partner’s face) than mutual (i.e., looking at one of the predefined ROIs on the partner’s face) looking behaviors. During mutual looking exchanges, the most time was spent in eye-to-mouth looking combinations; however, time spent in direct eye-to-eye contact was a significant predictor of the magnitude of gaze cuing, which was elicited by the gaze direction cues of interactive partners. The same positive relationship with individual gaze cuing magnitudes was not observed for non-mutual gaze looking. Thus, mutual iterative looks via eye-to-eye contact, rather than mutual looks via face-to-face contact, appear to be one of the vehicles for transmitting nonverbal social messages. We discuss three points related to these findings.

First, our data indicated that during interactions, participants were overwhelmingly more engaged in non-mutual relative to mutual looking behaviors, with non-mutual looks defined as those gaze patterns in which no reciprocal looks between the partners occurred towards any of the face ROI. These findings are surprising and do not align with the available literature on gaze exchanges in larger groups, such as three- or four-member groups^11,25^. There are two potential reasons for this discrepancy. One is that gaze to the surrounding environment may be moderated by group size^43^. Tree-and four-member group interactions allow for alternate roles in which group members can observe and contribute to ongoing gaze exchanges without engaging in direct eye-to-eye contact by, for example, looking at one group member while this person is looking at someone else^43,44^. Dyads, on the other hand, due to their simpler social structure, do not allow for similar relief from mutual looks. Therefore, participants in dyads may opt out of mutual looks in favor of not looking at the interactive partner at all in order to passively contribute or to disengage. Two, interactive looking may also be modulated by person familiarity and task demands. Our dyads were previously unacquainted individuals. And although we have used the survival scenario to stimulate their interaction, direct eye-to-eye contact is often considered intimate and physiologically arousing^45^ and thus may occur less frequently between unknown relative to familiar individuals^19^. The nature of the task may also have an effect. Here, the survival task required participants to verbally discuss an imaginary scenario. Mutual looking was not required. It is possible that in a different task, where mutual looking is encouraged with for example assigned leadership roles, the prevalence of looking behaviors may differ. Thus, future research may examine whether and how the prevalence of mutual looks changes with group size, familiarity of interactive partners, and the nature of the task.

Second, our data revealed that mutual looking behaviors involved looking at different regions of the face, at the eyes and mouth regions. Thus, mutual looks can occur as a general face-to-face contact, with possibly specific messages relayed in different combinations of looking patterns towards the top (eyes) and bottom (mouth) of the face. Participants spent the least amount of time engaged in direct eye-to-eye contact. This was surprising given the theoretical relevance of the eyes in social settings and the overwhelming amount of work illustrating the importance of eye-contact in communication^22,24^. Once again, this could reflect a relatively simple social nature of the dyadic interaction in which not engaging in direct eye contact may represent a way to disengage and/or passively contribute to the interaction (i.e., listen). Similar prevalence was found for mouth-to-mouth mutual looks, which also may be less common in the context of a dyadic interaction. While mouth region may be attended to derive speech related information^40^, this face part is also diagnostic for different emotional facial recognition, particularly for happy expressions and thus may be used to glean emotional content of the conversation^6,46^. Lastly, participants spent the most amount of mutual time engaged in eye-to-mouth interactive looking pattern indicating importance of different face parts to social communication, which as discussed may have been used to understand the conversation.

Finally, while mutual looks occurred in different combinations of looking, direct eye-to-eye contact was specifically related to the magnitudes of gaze cuing elicited by the images of the interactive partner’s gaze cues. This finding differs from past research. For instance, Capozzi and Ristic^25^ found that non-mutual looks (social referencing specifically, which refers to the time a member is looked at by other group members) was predictive of gaze cuing magnitude in three-member groups, such that gaze cues from participants who were looked at more by their interactive partners elicited a greater gaze cuing effect^25^. The same relationship was not found for direct eye-to-eye contact, or mutual looks. Hayward et al.^27^ also found that direct eye-to-eye contact experienced during a live interaction was not a reliable predictor of the magnitude of the cuing effect. However, in Hayward et al.’s study, gaze cuing was elicited by a schematic face rather than the face of an interactive partner. Thus, the relationship between the time spent in direct-eye-to-eye contact during real life interactions and later gaze following appears to depend on the utility of gaze cues to which the participants are responding (i.e., those cues experienced before). Further support for the link between eye-to-eye looking pattern and social communication in our data comes from the analyses of non-interactive looking exchanges in which looking at eyes in a non-interactive manner without reciprocation was not related to the magnitude of gaze cuing elicited in response to interactive partner’s gaze cues. Thus, it appears that the amount of time spent in eye-to-eye contact engages dual and interactive nature of gaze and as such may be key to relaying social messages between individuals.

The outstanding question about the content of social messages relayed by eye contact remains. One possible explanation for the relationship between the amount of direct eye contact and increased magnitude of gaze cuing is that the eyes communicate social messages such as intent^45^. Past work has demonstrated that direct eye contact is associated with activity in the brain regions associated with the detection of communicative intent and language^47^. Similarly, eyes have also been associated with communication of mental states and social status. Van Overwalle and Baetens found that the anterior rostral medial prefrontal cortex, an area of the brain associated with making inferences of mental states in social tasks was activated during mutual looking behaviors^48^. Furthermore, the eyes also communicate emotional states and interest. Cavallo and colleagues found that when dyads engaged in direct eye contact, they had increased skin conductance associated with arousal^47^. Hence, the context of the social messages communicated by the eyes remains a highly interesting research question for future work.

To sum up, the present study is one of the first to use dual mobile eye trackers during natural and unconstrained live interaction to investigate looking behaviors in dyadic interactions. This technology allowed us to examine the fine-grain nature of eye movements during interactions, and to specify the prevalence of different combinations of dyadic looking patterns. Our data showed that participants engaged in non-mutual looking behaviors relative to mutual ones more frequently. Further, mutual looks in the form of direct eye-to-eye contact occurred infrequently but was especially important in relating the gaze dynamics at the dyad level with individual functionality in gaze following, whereby the magnitudes of gaze cuing were larger for individuals who engaged in direct eye-to-eye contact more during mutual interactive exchanges only. As such, these data provide one of the first direct links between social communication via the eyes at the group level and individual social behavior and show experimentally that eye to eye contact is an important social vehicle in nonverbal communication.

## Data Availability

The datasets generated during and/or analyzed during the current study are available from the corresponding author upon reasonable request.
